# Environmental justice in South Africa: the dilemma of informal settlement residents

**DOI:** 10.1007/s10708-022-10808-z

**Published:** 2023-02-07

**Authors:** H. N. Kekana, T. M. Ruhiiga, N. N. Ndou, L. G. Palamuleni

**Affiliations:** 1 Gauteng Tourism Authority, 124 Main Building, Johannesburg, 2001 South Africa; 2 Department of Humanities Education, Faculty of Education, Kabale University, P. O. Box 317, Kabale, Uganda; 3 University of Fort Hare, 1 King Williams Town, P/Bag X 1314, Alice, 5700 South Africa; 4 School of Geo and Spatial Sciences, North West University, P/Bag X 2046, Mmabatho, South Africa

**Keywords:** Environmental justice, Informal settlements, Environmental impacts, Kosmos

## Abstract

The concept of environmental justice is well established in the literature; however, scholars still battle to agree on what it really means. This concept has become more relevant to the studies of informal settlements amongst others. The location and environmental variables in informal settlements suggests a variety of injustices that comes with location, limited access to water, poor or lack of sanitation, challenges with transport availability, accessibility, affordability, and lack of other social amenities. These and many other socio-economic needs forms part of the value chain of environmental justice debates across the world. This paper deals with environmental justice in the informal settlements of Kosmos, in the Madibeng Local Municipality, Bojanala Region in the North-West Province of South Africa. The paper highlights some of the environmental challenges faced by the informal settlement residents such as pollution, waste management (landfill sites, waste collection) sanitation and water provision. The paper explores how the Kosmos informal settlement community has been excluded from decision making processes regarding their own environment and considers the levels of environmental injustices commonly associated with this kind of practice.

## Introduction

Environmental Justice is frequently presented as a relatively new concept worldwide (McGregor, 2009). Organisations, environmental activists, and leaders continue to call for environmental justice for communities living under squalid conditions, against mining and other industrial companies that pollute the air and fresh waters, and against the general living conditions of the poor. In South Africa, the term “Environmental Justice” found its first concrete expression in 1992 at a conference organised by Earthlife Africa (McDonald, 2003). The conference brought together leading South African Environmentalists and academics from around the world to map out the future of the environmental justice movement in South Africa (McDonald, 2003). This conference, and many others around the world, prioritised environmental challenges faced by the poor and exposed inequalities in the distribution of services especially water and sanitation. This inequality persists today especially in informal settlements, where one community will have services while an adjacent one will not have.

In the South African context, environmental justice means social transformation directed at meeting basic human needs and rights, and a central idea in a nascent grassroots movement which is fuelled by the growing contradiction between the discourse of rights and the experience of unmet needs (Cock, 2004). While there is no agreed definition on what environmental justice really means, this paper deals with this concept in terms of geographic associations, and focusses on pollution, waste management (landfill sites, waste collection) sanitation and water provision as common variables that affect the informal settlement environments using the Kosmos informal settlement as a study area. Justice in this regard, will refer to the role of authorities in addressing environmental challenges faced by the community of Kosmos informal settlement in the Madibeng Local Municipality, Bojanala District, North West Province, South Africa.

## Literature review

Environmental justice (EJ) has been a central concern in a range of disciplines, and both the concept and its coverage have expanded substantially in the past two decades (Schlosberg, 2013). According to Khosravaninezhad and Akbari (2014), Environmental Justice (EJ) concept consists of multifaceted movements, community struggles, and discourses in contemporary societies that seek to reduce environmental risks, increase environmental protections, and generally reduce environmental inequalities suffered by the minority and poor communities. The writer further maintains that the term incorporates ‘environmental racism’ and ‘environmental classism,’ which captures the idea that different racial and socioeconomic groups experience differential access to environmental quality. Scholars also argue that racism plays a critical factor in environmental planning and decision-making processes in the US and other settler nations (Parsons et al., 2021).

Defining EJ as a concept with racial connotations resonates with the reality of many indigenous communities around the world and is true to countries like South Africa wherein black people are still subjected to poor housing, lack of land, water, sanitation, and generally poor inhabitable places that they call home. Despite how well-intended the EJ scholarship is, the dominant EJ framework being used by scholars (and applied to indigenous communities around the globe) continues to neglect the unique experiences of indigenous communities and their collective trauma under colonialism (Whyte, 2016). Over time, scholars turned to agree about the need to extend the environmental justice concept to accommodate other realities that fits within this notion. Extending the environmental justice framework, which has had limited theoretical rigor, to other geographic and cultural contexts has facilitated a deeper understanding of environmental justice as an evolving and expansive concept (Ranganathan & Balazs, 2015).

Holifield (2013), defined the term environmental justice as geographic associations between pollution or waste sites and low-income or minority communities. The author also acknowledges that the researchers’ discourse continues observed patterns, with no consensus on what constitutes inequality and injustice. Many grassroots activists insist that environmental justice demands the prevention of all forms of toxic pollution (Holifield, 2013). For some, environmental justice means access to water (McDonald & Jones, 2018), sanitation (Winter, 2017), and housing. For example, in Cape Town South Africa, a recent drought documented by Jehanzaib et al. (2020), threatened the water supply of 4 million residents, many of whom live in the city’s sprawling informal settlements where access to water services is much more unreliable than in the more affluent areas (Enqvist & Ziervogel, 2019). This is one example of how environmental justice is defined in the provision and availability of fresh drinkable water for the people of Cape Town. In a study of environmental justice in India, Whyte (2011) argued that different views exist on the moral basis of standards of environmental justice. For some, environmental justice is seen as a policy matter (Mehta et al., 2014), while others view EJ as a social movement, and a call to equal access to the decision-making process (Khosravaninezhad & Akbari, 2014).

Schlosberg (2013) mentioned four dimensions of EJ as distribution, recognition, procedure, and capability. The writer maintains that the distribution of toxics and hazardous waste in the United States was the original focus of distributive justice, and a focal point in the EJ debate. Most of the environmental justice movements around the world have emphasized the need for recognition and their capability to raise and address environmental injustices in many ways and form. Part of this concern was headed in their participation at the 2011 Conference of the Parties (COP 17) held in Durban South Africa. Recognising the rights of communities and their ability and capacity to speak for themselves, is very important in the environmental justice debates. Recognition is also expanded in this paper to include responsibilities of individual human activities towards the environment. We can no longer talk about EJ outside the fiducial responsibilities of human and community activities.

For example, in most communities, there are procedures to be followed in waste disposal. Within the connotations of EJ, the question is whether individuals within communities take responsibility and follow these procedures. This is also a question of education, capability, or willingness to do what is correct to safeguard their own environment. A capabilities approach to justice, which encompasses a range of basic needs, social recognition, and economic and political rights, has offered a broad framework with which we can understand the array of demands of environmental justice movements (Schlosberg, 2013).

Environmental justice is linked to a diversity of variables with climate and land use change being at the centre. Global climate change threatens where and how people live (Siders & Ajibade, 2021). Mohtat and Khirfan (2021) talks about ‘climate justice’ as both a social equity concept and practical process for action research. Because of climate change, coastal areas and communities around the world will be increasingly impacted by diverse hazards including sea-level rise, flooding, and eroding shorelines, leading to increasing displacement of people (Tubridy et al., 2022). Climate change and its effects such as frequent and intense storm surge events, rising water tables and rising seawater levels in coastal areas have worsened the situation (Apraku et al., 2018; Ofosu et al., 2020; Ziervogel, 2019) cited by Membele et al., (2021). These are common challenges faced by communities living within the low-lying coastal areas, flood plains and riverbanks, and are practical examples of environmental justice challenges around the world.

Kemper et al. (2015) argued that, understanding the dynamics of human settlements is a pre-requisite for sustainable development and environmental management. The proliferation of informal settlements has become the norm in South Africa and their frequency is undetermined. Extensive literature has covered the growth, risks, health, and environmental hazards accompanying the formation of informal settlements in cities and urban areas, however, there are still gaps in understanding the daily environmental challenges that informal settlement communities deal with and the environmental justice challenges that prevails in this type of habitat. There has been a general focus on poverty, unemployment, the inability of informal settlement communities to meet daily needs, housing provision and settlement upgrading programmes, as important measures of informal settlement dwellers’ sustainability. These and many others, are crucial environmental habitat success drivers and researchers should consider their effects on the surrounding open land, air, and underground environments in relation to their impact on environmental justice.

A critical issue is that many informal settlements occupy land that is unsuitable for development, which has resulted in the destruction of environmentally sensitive areas (Aguilar & Santos, 2011). In many cities, the informal occupation of areas near water reservoirs, areas prone to landslides and flooding, or protected forests is another looming problem (Fernandes, 2011). Most illegal occupations, spontaneous or organised, are occurring in inadequate or high-risk areas such as at the margins of small streams and their headwater areas, deactivated mining areas, below transmission lines or along recently constructed highways (Zeilhofer et al., 2008). Williams et al. (2019) cited by Membele et al. (2021), said that, in South Africa, people in informal settlements are vulnerable to flood hazards because they live in hazardous areas and have poor socio-economic conditions. It must be acknowledged that most of the threats are related to locations where housing or services are inadequate (Nasser & Elsayed, 2018). This is part of the “justice” debate and is at the centre of inequalities that informal settlement communities find themselves in.

The ecological implications of these tendencies have been a source of enormous concern due to ecological degradation caused by the persistence of informal settlements lacking basic services (sewerage, water, waste disposal) or ineffective policies for protecting ecosystems (Aguilar & Santos, 2011). Additionally, informal settlements are associated with high levels of poverty, illiteracy and crime, inadequate local services, especially healthcare, education, and youth facilities (Nassar & Elsayed, 2018). Fernandes (2011) noted that informal settlements and development have generated fragmented cities and precarious neighbourhoods, profoundly marked by many forms of health and safety hazards, environmental degradation, pollution, and inadequate sanitary conditions; often associated with narrow streets, dense occupation, precarious construction, difficult access and circulation, lack of ventilation, sanitation, and public spaces.

## Materials and methods

The research focused on qualitative data with categorically identified variables in the study site. The research used a questionnaire, interviews, and observations methodologies. The aim was to reveal human experiences, and their individual opinions in relation to the identified and targeted human activities. The use of these methods was deliberate and provided the study with a wider opportunity to gather as much relevant information as possible to identify environmental justice as an important factor in the lives of people living in informal settlements.

### Description of the study area

**Fig. 1:**
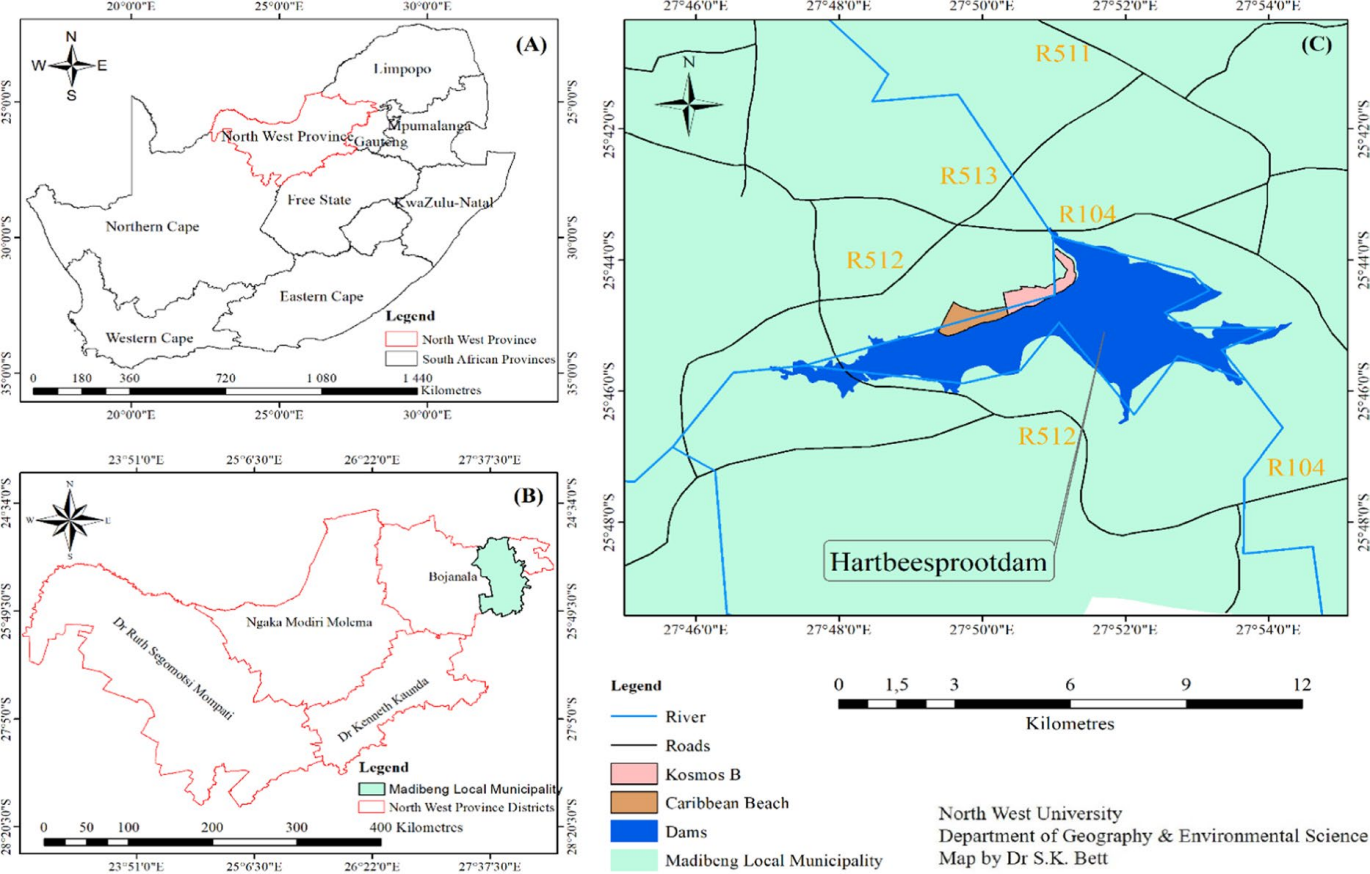
Map of the study area. *Source*: North West University, Department of Geography and Environmental Science

The study area encompassed the Kosmos informal settlement located north of the Haartbeespoortdaam along the Simon Berker Avenue that leads into the Kosmos area (DHS, 2006) in Madibeng Local Municipality, North-West Province, South Africa (Fig. 1). The surrounding area is mainly residential land with both the Kosmos Ridge, Carrabean, Mount Kos, and Kosmos villages located within a 1 km radius. Access to the area in which the informal settlement is located is from the R512 Route located north-west of the settlement (DHS, 2006). Kosmos informal settlement includes an estimated population count of about 886 residents (StatsSA, 2011). At the time of this publication, the results of the Census 2022 were not yet available as Census 2022 data was still undergoing assessment. This population estimate might have changed over time due to continuous in and out internal migration. The Kosmos informal settlement has a mixed population with most of the people coming from neighbouring countries such as Zimbabwe, Mozambique, and Malawi.

In Madibeng Local Municipality there are 45 informal settlements spread across the municipal area. The choice of Kosmos informal settlement as a study area for this paper was informed by its unique location within a rich square mile of up-market residential suburbs which made the issue of environmental injustice more pronounced. The study site was selected based on its diversity in terms of growth history and the characteristics of the local environment. The site also reflects a diverse typology, residential outlook, different social and economic setup, and density, and above all, its relevance to the environmental justice debates. The following map (Fig. 1) presents the location of Kosmos Informal Settlement.

The Integrated Development Plan (IDP) of Madibeng Local Municipality presents Kosmos informal settlement as an illegal squatter settlement. Residents of the surrounding up-market homes took the informal settlement community matter to court to have them removed from Kosmos and relocated. The court ruled in their favour, however, the community appealed the order. The Madibeng Local Municipality was cited as a second respondent and was ordered by the court to seek alternative land and remove the informal settlement. The municipality identified an area which is about 25 km away from Kosmos to relocate the community. Some community members opted to relocate while the majority remained behind with no intention to move out of Kosmos. Amongst the reasons cited by residents of the up-market homes is that the informal settlement has devalued their residential properties. This is a typical environmental justice matter which deals with locational advantage for the few. There is also a silent racial discrimination practice at play because the informal settlement is predominately occupied by black Africans while the upmarket homes belong to majority white residents.

### Sampling and sample population

Given the heterogeneous nature of informal settlement communities, the study opted for a stratified random sampling technique using the population documents obtained from DHS and StatsSA. Members of the targeted community (population) were divided into three homogeneous age groups (strata) namely: 0–20 years, 21–40 years, and 41–60 years. Questionnaires were administered to randomly proportionately selected participants from each age group yielding 90 respondents from the study area (“Appendix 1”).

### Data collection

A questionnaire was used to collect data on the following aspects: the demographic and social backgrounds of respondents, the origins of the settlement and background historical locations, duration of stay in the area, perspectives of informal settlers on services, socio-economic conditions, land use and environmental management and perceived or actual impacts of land use changes. The aim was to reveal human experiences, and their individual opinions in relation to the identified and targeted human activities. The use of these methods was deliberate and provided the study with a wider opportunity to gather as much relevant information as possible. The data were supplemented and verified by secondary data obtained from the Department of Human Settlements (DHS) and Statistics South Africa (StatsSA). The DHS data included population, household information, maps, water, and sanitation levels of service (LOS) for Kosmos. In addition, observations helped to identify silent but visible activities within the Kosmos informal settlement study area.

### Data analysis

For this study, data from questionnaires was analysed using the XLSTAT data analysis tool. XLSTAT is a suite of statistical add-ins for Microsoft Excel developed in 1993 by Addinsoft to enhance the analytical capabilities of Microsoft Excel. The analysed demographic and socio-environmental characteristics of the respondents and the study areas have been presented in tables as frequencies and percentages.

## Results

Environmental justice concerns in Kosmos, are characterised by the inadequate provision of energy resources, risks of the communities to pollution, inadequate waste management services, and susceptibility to water related diseases due to lack of water and sanitation services. Each variable is explained, and results thereof captured respectively.

### Deforestation

**Fig. 2:**
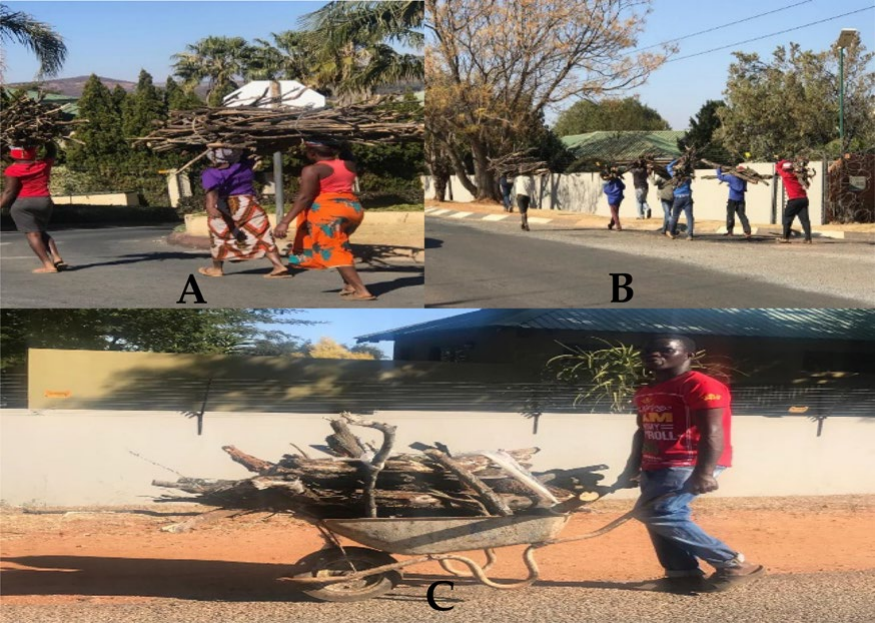
Collecting firewood in Kosmos informal settlement. *Source*: Author

The Kosmos informal settlement does not have electricity, and the community relies on firewood and paraffin fuelled stoves for household water heating and cooking. Collection of firewood is a daily hustle for the community of Kosmos informal settlement, with twice weekly collections necessary to meet their energy demands. The use of firewood is prevalent within the Kosmos informal settlement and contributes to the high levels of deforestation in and around the area. The level of deforestation in the area is pronounced with encroachments into the greenbelt of Hartbeespoortdaam area along Leloko towards Hekpoort. Figure 2A–C taken during the field work phase shows residents from Kosmos on their way from fetching firewood.

Carrying firewood on the head (Fig. 2A, B) is a traditional practice and continues to date as the most economic means to transport firewood, albeit with all its health risks such as skeletal neck and back problems associated with carrying heavy loads (Evans et al., 2013). Both women (A) and men (B and C), collect firewood indiscriminately daily. The residents of Kosmos travel long distances (sometimes up to 6 km) on foot in search of firewood, ferrying it on their heads or in wheelbarrows (Fig. 2C). Lack of electricity and heavy reliance on fuelwood energy presents a form of environmental injustice issue to the Kosmos informal settlement community, while their immediate neighbours in the up-market homes have electricity as energy source. This is a clear sign and practice of environmental injustice between the two adjacent communities.

**Fig. 3:**
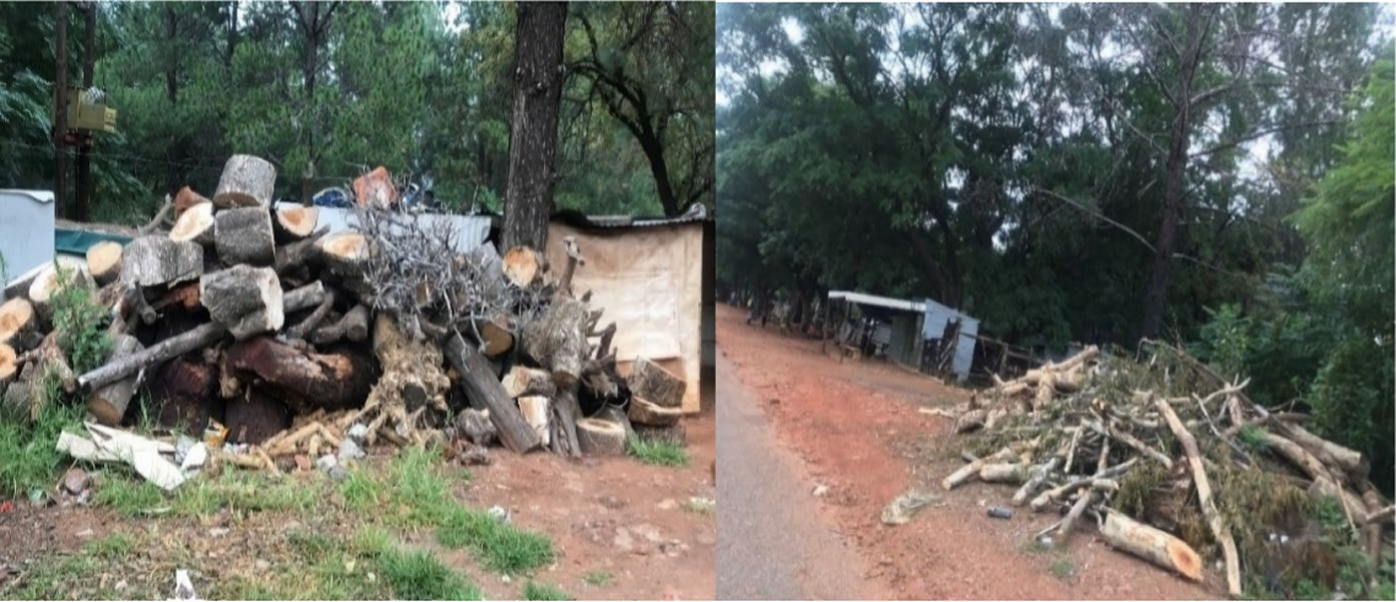
Piles of firewood in Kosmos informal settlement. *Source*: Author

Some community members in the Kosmos informal settlement collect and sell firewood to generate income, collecting two to three loads per day. A load of wheelbarrow retails between R50 ($3.15) and R100 ($6.28). Figure 3 shows two piles of firewood along the main road in the Kosmos informal settlement.

**Fig. 4:**
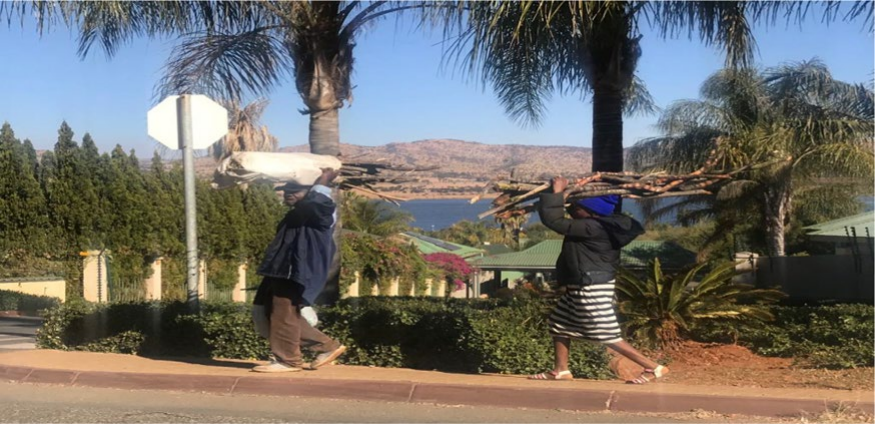
Woman carrying firewood (Kosmos informal settlement). *Source*: Author

Figure 3 shows piles of firewood that were cut using a saw cut machine. The use of electrical machinery to cut trees for firewood has become prevalent in the area, especially for commercial purposes. The firewood is piled outside the informal settlement for greater visibility to potential customers, and lack of internal roads with truck or tractor makes delivery access difficult. The Kosmos informal settlement represents a settlement contradiction because of its location inside a rich square mile of private residential estates. Figure 4 highlights this contradiction and shows woman carrying firewood, passing an electrified upmarket Carrabean housing estate to the right with part of Hartbeespoortdaam water body visible in the background. Residents of the estates enjoy having electricity as an important source of energy while those living on the other side are subjected to the consequences of environmental injustice exposing them to preventable diseases due to lack of electricity. This is a clear illustration that some communities’ experiences more environmental risks than others (Schlosberg, 2013).

### Pollution

Pollution is a common problem in informal settlements, and at the centre of environmental justice debates worldwide. In the Kosmos informal settlement, air pollution is prevalent due to the use of firewood and paraffin fuel sources for cooking and heating. The fumes from firewood and paraffin stoves results in high levels of air pollution which affects the immediate and surrounding areas and can potentially cause respiratory disorders especially amongst children. Environmental injustice contributes to higher environmental health threats emanating from particulate matter emissions among the informal settlement residents. As mentioned by Olaniyan et al. (2017), various studies have demonstrated that exposure to ambient air pollutants in early childhood triggers asthmatic attacks and exacerbates other respiratory symptoms. However, the evidence for the extent to which air pollution affects children’s respiratory health is inconclusive suggesting the need for further investigations (Olaniyan et al., 2017). During the winter season, the informal settlement is covered by a dark cloud of smoke from the extensive use of firewood for heating purposes. This causes air pollution which is one major environmental hazard, and the smoke extends to the private residents. This is one of the reasons that the private homeowners within the area took the informal settlement community to court to get them evicted from the land.

**Fig. 5:**
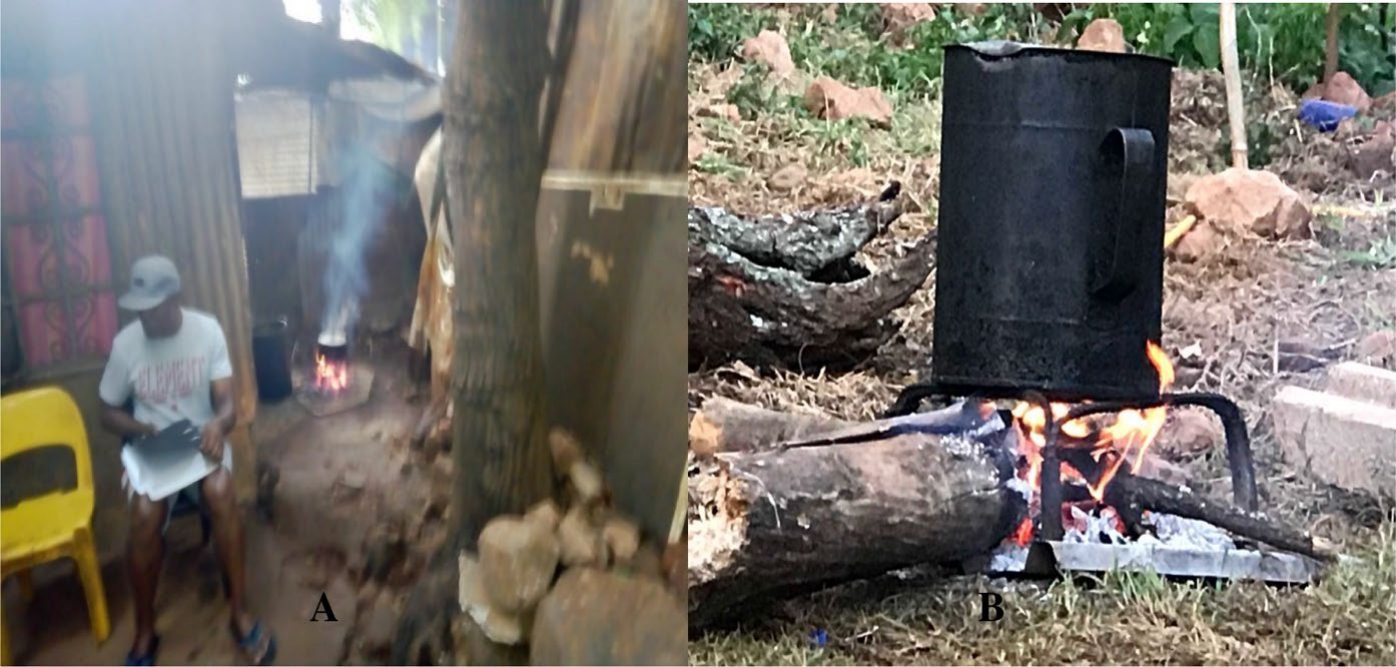
Use of firewood for cooking and heating. *Source*: Author

Figure 5a shows smoke emitted from firewood cooking during the day. The smoke not only cause air pollution, but gets trapped within the shack building, leaving the shack with lingering residual smoke during daytime and night-time. Figure 5b shows the use of firewood to boil or heat water during the day. The fireplace is a few meters from the shack, allowing smoke diffusion into the surroundings further contributing to atmospheric air pollution.

Most of the shacks do not have windows and this presents a health hazard and poses high risks for respiratory diseases due to poor ventilation and limited airflow circulation inside the shack. Barbieri et al. (2017) mentioned an important but neglected part of human habitats within informal settlements, cooking technologies and their impacts on the socio-economic and environmental perspectives. The writers argued that the humanitarian response usually focuses on food availability and access, while food processing is often neglected, and in this framework, cooking technologies play an essential role. Unsustainable and inefficient cooking technologies or practices can have direct impacts on food preparation, and indirect effects on local biomass resource overexploitation and the health of local people and communities (Barbieri et al., 2017). Cooking technologies or means of food preparation, have been common avenues of environmental toxicants in informal settlements, especially the use of firewood, coal, charcoal, and paraffin stoves in poor ventilated environments. Due to continuous exposure to contaminated air, Kosmos informal residents face more health problems, and this presents a high level of environmental injustice to the community.

### Waste management, waste collection and landfill sites

**Fig. 6:**
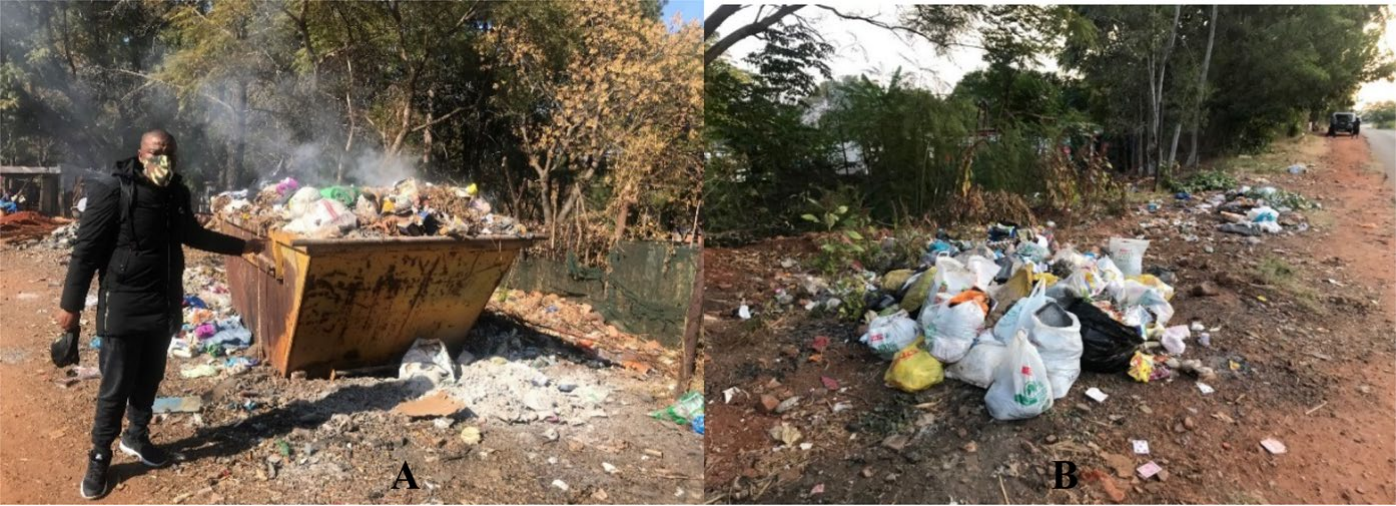
**A** Waste skip bin. **B** Waste overflow outside skip bin. *Source*: Author

The Kosmos informal settlement has a landfill site situated about 1.5 km from the informal settlement. There is also one waste collection skip bin as shown in Fig. 6A provided by the Madibeng Local Municipality. The skip bin is positioned at the side along the informal settlement’s main road. This is the most feasible location for the skip bin due to lack of access roads into the settlement. The municipality is supposed to empty the skip once a week, but collection has not been happening as frequently, with rubbish overflowing and people dumping waste around the skip bin (Fig. 6A). Figure 6B shows waste overflow on the ground next to the skip bin.

The community incinerates the waste in the skip bins to manage the volume of waste so that they can continue to use the skip bin. Unfortunately, the burning of waste also damages the skip bin due to excessive heat trapped inside it. During windy days, the waste scatters in and around the informal settlement and onto the main road. Due to the lack of an effective waste management plan, the informal settlement is littered with waste, and can potentially cause airborne and other communicable diseases. Zapata and Campos (2014) argued that informal settlements in the global South cities are often neglected by formal solid waste collection services. This is a reality within the Kosmos informal settlement with visible disparities between the two communities (Kosmos and Carrabien Estate) disproportionately serviced by the same municipality. Environmental injustices contribute to the disparities in waste collection by the municipality whereby the informal settlement can go for days without refuse collection while the private residential estates get the same service on a weekly basis, albeit being 200 m apart. This is another clear example of what Schlosberg (2013) refers to as social injustice.

In fact, the refuse removal trucks from Madibeng Local Municipality drive past the Kosmos informal settlement to collect waste from private households, leaving the informal settlement waste bins overflowing. The impact of environmental neglect by the local municipality, considering the Kosmos informal residents as undeserving to waste removal compounds the greater risk of environmental nuisance emanating from uncollected waste. From this perspective, environmental justice alluded to waste collection and management is seen as a legitimate environmental health issue which deserves greater attention from the Madibeng Local Municipality. Mehta et al. (2014) mentioned that this is due to the contradictory nature of the state and its disregard for marginalised people, unequal experiences of citizenship, elite biases in policy making and planning, resource capture by powerful players as well as significant distributional, recognition and procedural problems.

**Fig. 7:**
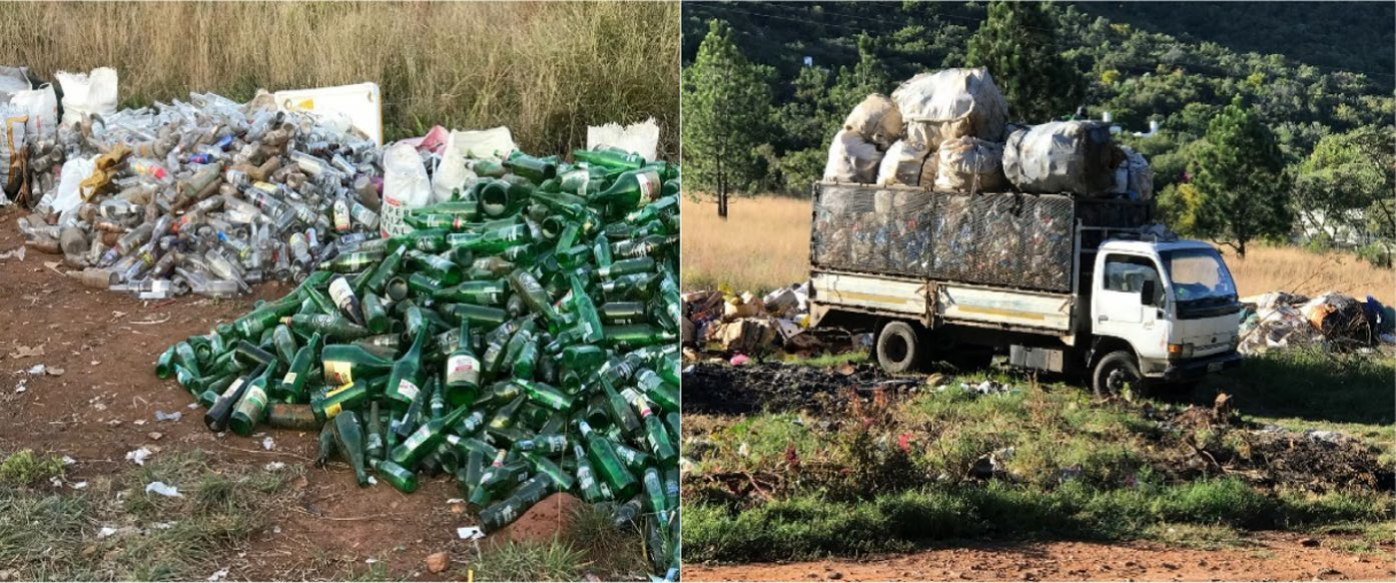
Waste recycling (Kosmos informal settlement). *Source*: Author

To challenge the environmental injustice of uncollected waste, some members of the community started a small-scale waste collection and recycling business of plastic and glass bottles. This is part of the attempts by what Gutberlet et al. (2016) refer to as ‘social entrepreneurship’. These are individuals who collect and recycle waste for a fee as part of their source of income and means of survival as seen in Fig. 7.

Gutberlet et al. (2016) attest that waste pickers in the informal sector represent one of the most widely excluded, impoverished, and disempowered segments of society and are exposed to toxic materials, suffer from prejudice and stigmatisation. Most often, the waste pickers experience difficulties in creating formal cooperatives or associations, lack access to official microfinance and funding opportunities, are susceptible to price market oscillations, and are subject to exploitative relations with intermediaries (Gutberlet et al., 2016). Most of the recycled bottles in Kosmos are from beer and other alcoholic beverages, a common challenge in informal settlements. There is a high level of alcohol abuse in the area, and this often leads to social instability, fights, and stabbings. Social justice is highly impacted in this community and there is need to improve their living conditions to impact positively on their livelihood.

### Water provision

According to the Water Services Amendment Act 30 of 2004 (Act 30 of 2004), all citizens in South Africa must have access to safe, clean, quality drinking water, and basic sanitation amongst other things. The Act recognises the right of access to basic water supply and sanitation services necessary to ensure sufficient water access and a safe environment, not harmful to residents and their well-being (DHS, 2006). The Act further acknowledges that all spheres of government must strive to provide water supply and sanitation services sufficient for subsistence and sustainable economic activity (Act 30 of 2004). The DHS (2006) report shows the level of service definition for water and sanitation based on the Norms and Standards for Quality Water Services as outlined in the Water Services Act (Act No. 108 of 1997) (DHS, 2006). Table 1 was adapted from the DHS (2006) report, and it shows the level, code, and description for water provision.

**Table 1:** Level, code and description of water provision services. *Source*: Department of Human Settlements (DHS, 2006)

Level of service definition (water provision)		
Level	Code	Description
Low level of service (below minimum basic standards)	LLOS	(1) Potable water sourced from storage tanks/communal standpipes located beyond 200 m walking distance
		(2) Potable water supply of less than 25 L per person per day
		(3) Consumer supplied with potable water for less than 7 days in a year
Intermediate level of service (meeting minimum basic standards)	ILOS	(1) Potable water sourced from storage tanks/communal standpipes located within 200 m walking distance
		(2) Potable water supply of at least 25 L per person per day
		(3) No consumer is without water for more than 7 days in a year
Full Level of Service	FLOS	House/Yard connection supplied through a regional water distribution network

The Kosmos informal settlement’s water provision infrastructure is provided and serviced by the Madibeng Local Municipality. The water infrastructure is reticulated and provided through standpipes in between the shacks. Based on Table 1, the Kosmos informal settlement can be categorised under the Full Level of Service (FLOS) (DHS, 2006). The DHS (2006) report further indicates that the Kosmos informal settlement is situated within the 1 km buffer of a major water body, the Haartbeespoortdaam. Their water connection is part of the main water source for private residents within the Kosmos area, and they seldom experience water supply disruptions. The water reticulation systems fill their water lines first, before entering Mount Kos, Falcon View, and Kosmos Village private residential properties. This is by pure coincidence that the informal settlement is located between the up-market residential properties, as such, some level of justice prevails in terms of water provision in this regard. The challenge remains consistent, and uninterrupted supply of water.

### Sanitation

Despite many recent policies and interventions to reduce the number of people without access to sanitation around the world, 2.4 billion people are still living without a toilet (WHO/UNICEF, 2015) cited by Winter (2017). Poor sanitation is a serious public health issue and a violation of people’s human rights (Acharya et al., 2015; UN General Assembly, 2010). When human rights are violated, justice cannot prevail, and in this case, environmental justice is at stake. Winter (2017) further noted that while access to sanitation is a global issue, there are large disparities in access across different regions, countries, and social and geographical contexts. Lack of access to sanitation is a persistent problem in sub-Saharan Africa, where less than 20% of the current population has access to sanitation (WHO & UNICEF, 2015). Environmental justice demands sanitation provision as part of human rights.

The problem of poor sanitation is also a particularly critical issue for people living in informal settlements, where high population densities combined with a deficiency of sanitation services makes it difficult for residents to avoid contact with human waste (Winter, 2017). The South African Water Services Act (Act No. 108 of 1997) provides for basic sanitation that is not harmful to the environment (DHS, 2006). According to the 2014 Municipal Integrated Development Plan (IDP), 51% of Madibeng Local Municipality households do not have access to basic sanitation services (DHS, 2006). Of those households that have access to water borne sanitation, 83% are being serviced by the Madibeng Local Municipality, mainly at the Brits, Mothutlung, Rietfontein and Letlhabile Wastewater Treatment Works, with the remaining 17% being serviced through private package plants (DHS, 2006). Table 2 shows the level, code, and Level of Service Definition for sanitation from the study area.

**Table 2:** Level of services for sanitation. *Source*: Department of Human Settlements (DHS, 2006)

Level of service definition (sanitation)		
Level	Code	Description
Low level of service (below minimum basic standards)	LLOS	A pit latrine that is not safe, reliable, environmentally sound, easy to keep clean, does not provide privacy and protection against the weather, is not well ventilated, does not keep smells to a minimum and prevents the entry and exit of flies and other disease-carrying pests
Intermediate level of service (meeting minimum. basic standards)	ILOS	A pit latrine that is safe, reliable, environmentally sound, easy to keep clean, provides privacy and protection against the weather, well ventilated, keeps smells to a minimum and prevents the entry and exit of flies and other disease-carrying pests e.g. VIP toilet
Full level of service	FLOS	A waterborne sanitation system that is safe, reliable, environmentally sound, easy to keep clean, provides privacy and protection against the weather

Kosmos informal settlement uses pit latrines which is a sanitation level below the minimum basic standard (the LLOS level, Table 2). This is a violation of environmental justice and a serious health hazard because access to sanitation has a direct bearing on the right to human dignity (Saleem et al., 2019). There are few pit latrines in the entire settlement, which are meant to be used by multiple households, leading to potentially serious environmental hazards, airborne and high risk of the spreading of infectious diseases. More alarming is that most of the pit latrines were built using a combination of plastic, wooden boards, and steel corrugated sheets.

Hildebrand and Corburn (2015), concluded that, “inadequate urban sanitation disproportionately impacts the social determinants of women’s health in informal settlements and, the impacts on women’s health include infectious and chronic illnesses, violence, food contamination and malnutrition, economic and educational attainment, and indignity”. In essence, lack of access to sanitation privacy is a cry for justice especially for vulnerable groups such as women and children who are exposed to danger when using a sanitation facility that is not private. The health of women often correlates with the health of children and the health of communities in general, since many women living in urban informal settlements disproportionately support economic and community activities (Hildebrand & Corburn, 2015).

The Madibeng Local Municipality once provided the Kosmos informal settlement community with portable bucket toilets. Typically, the community would use the bucket toilets and the municipality would collect, empty, and clean the toilets weekly. Servicing this bucket toilets never happened and all the toilets provided got filled up. Some members of the community decided to empty and clean a few bucket toilets for continuous use, but this has also not been sustainable. Children in the Kosmos informal settlement use the back of pit latrines for fear of falling inside the pit latrines. When it rains, water collects human waste from around the pit latrines and washes it downstream. Some human waste also flows into the informal settlement homes violating the environmental rights of some residents.

The situation in Kosmos area is complicated because the area is very dry and rocky with hard topsoil surfaces which impedes water drainage. Therefore, when there are heavy rains, water logging and flooding of pit latrines occurs causing environmental hazards, exposing residents to potential water borne diseases. The sloping scale of the land in Kosmos provides for high contamination of groundwater. This presents serious implications of environmental injustices that could arise because of potential groundwater contamination from the use of pit latrines. As mentioned by Okurut and Charles (2014), sanitation improvement is crucial in saving lives that are lost due to water contamination.

**Fig. 8:**
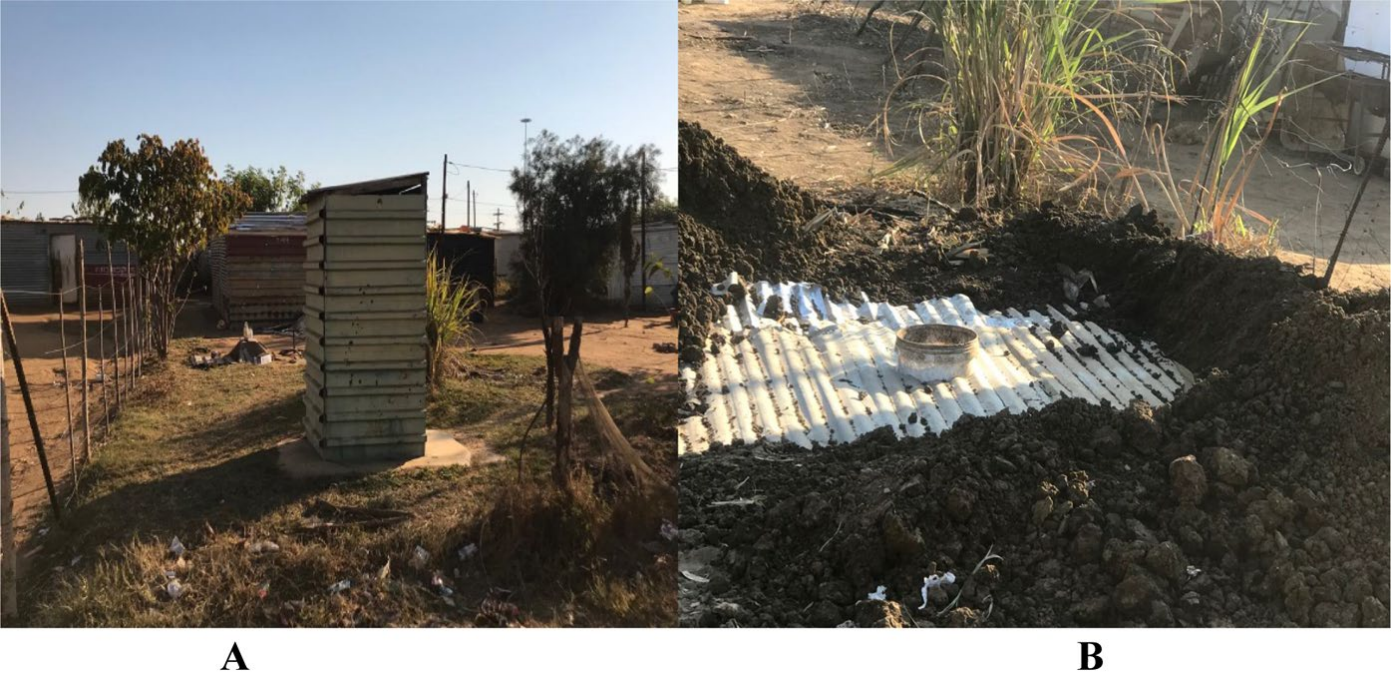
**A** Corrugated pit latrine. **B** Pit latrine preparations. *Source*: Author

Digging pit latrines inside the yard is common in informal settlements. Figure 8A is a complete pit latrine building with corrugated sheets. This type of structure is very common in informal settlements and is sold as a complete structure from hardware stores.

Figure 8B shows a newly dug pit covered with an iron sheet with a bucket going through a central hole on the corrugated sheet. The principle here is to cover the sheet with soaked mud or cement concrete and complete it with a corrugated structure like the one in Fig. 8A. Each household has a pit latrine and as they get full, they dig new ones next to the old structures. Pit latrines and the use of bucket systems for sanitation remains an environmental hazard and a serious injustice for the Kosmos community.

Understanding the demand for improved sanitation in the local context is critical if facilities are to be continually used (Okurut & Charles, 2014). The understanding with the bucket system was that the municipality would periodically return to the sites, collect, empty, clean, and chemically disinfect the bucket toilets and surroundings. However, this has not been happening in Kosmos informal settlements. The buckets stay full for extended periods without being serviced. This is a serious environmental injustice for the Kosmos informal settlement community. Resources are being wasted on installing facilities that are later misused or never used because they do not meet the local demand (Okurut & Charles, 2014). The use of pit latrines can cause airborne diseases and affect the community especially children. Environmental justice is about social transformation directed toward meeting human basic needs and enhancing the quality of life, a situation which is a distant dream for the residents of Kosmos. However, it was beyond the scope of this study to explore the extent to which differential sanitation risks contribute to higher rates of morbidity and mortality among the residents in Kosmos.

## Discussion

Environmental justice as a discourse has rapidly expanded its influence and has been applied to both a broadening range of issues, and, increasingly, at a global level (Schlosberg, 2013). This paper highlighted a range of environmental “injustices” faced by communities in Kosmos informal settlements. Deforestation, pollution, sanitation, waste collection, waste disposal, lack of landfill sites, and electricity were identified and presented amongst others, as common environmental injustices in the Kosmos informal settlement. Deforestation remains serious in Kosmos, with a large share of its natural forest lost because of firewood collection. Pollution was identified as a common problem, and it ranges from air pollution, due to the use of firewood, paraffin, and coal for energy, to underground water pollution from the use of sub-optimal pit latrines and uncollected waste buckets. Most informal settlements do not have access to electricity, and residents rely mainly on firewood and paraffin stoves for heating and cooking. These energy sources not only cause air pollution but pose greater risks for respiratory diseases and shack fires. Firewood consumption in Kosmos is higher than that of paraffin and coal due to the wider availability and relatively lower cost of the former.

Olaniyan et al. (2017) argued that paraffin use for cooking/heating is associated with susceptibility to passive smoking posing significant risk factors for adverse asthma outcomes. Paraffin use was associated with a twofold increased likelihood of having significant airway inflammation (Olaniyan et al., 2017). Paraffin and electricity were the most common energy sources in informal settlement communities in South Africa, and in many other low- and middle-income countries, among communities of low socioeconomic standing (Olaniyan et al., 2017). Similarly, Makonese et al. (2016), noted that informal settlements predominately use combustion fuels such as coal, wood, and paraffin to meet their domestic energy needs. The use of firewood and paraffin presents fire hazards as seen in many informal settlements across South Africa. Walls et al. (2019), maintains that in South Africa, the problem of fires in informal settlements is significant. The use of coal was not widely reported in Kosmos owing to its scarcity in the area, although it remains an alternative and available fuel source for heating and cooking.

The inadequate provision of sanitation services in informal settlements is generally worse than that of water and electricity services (Narayanan et al., 2017). Takem et al. (2009) concurs stating that, “the sanitation and water supply services are often inadequate in cities in developing countries.” Dwellings in informal settlements have no access to proper sanitation, and are exposed to indoor and outdoor polluted water that adversely impacts their health (Yuen, 2007). This statement supports the findings in Kosmos where leaking water pipes present the risk of water contamination. Inadequate drainage of storm water, greywater, and sewage plagues informal settlement dwellers throughout the developing world (Jiusto & Kenney, 2016). The dilemma to innovate and implement drainage solutions in informal settlements is further exacerbated due to the following physical challenges: densely packed shack homes, minimal open spaces, and social challenges associated with the often contentious, turbulent, and legally uncertain nature of informal settlements (Jiusto & Kenney, 2016). This statement supports the findings in the Kosmos informal settlement where narrow passages and no internal access roads for service vehicles were observed. Lack of access roads is a serious environmental injustice and community members walk a distance to the main road as they cannot access facilities and emergency services such as ambulances within their locality.

Informal settlements present a range of challenges from sanitation provision, including low incomes, insecure tenure, low education levels, difficult topography, and transitory populations (Okurut & Charles, 2014). In their study of sanitation services in Cape Town (South Africa), Mels et al. (2009) concluded that the main barriers to the implementation of proper sanitation systems were the non-permanent status of the informal settlements, high service maintenance costs, and their unsuitable location. Despite these challenges, Evans and Tremolet (2010) concluded that sanitation interventions need to address the local demand to ensure that facilities built are used to realise their full public health benefits.

Isunju et al. (2016), argued that informal settlements pose a high risk of spreading communicable diseases like cholera and dysentery due to poor sanitation conditions and overcrowding. The lack of sanitation services plays a major part in the spread of diseases (Napier, 2007). Solid waste collection, landfill sites, and waste disposal remains disproportionate and unsustainable within the Kosmos informal settlement. Ogwueleka (2009), cited by Maiyaki et al. (2018), concluded that solid waste needs to be appropriately managed to ensure general human wellbeing and environmental safety. Despite the growing awareness of the potential threat that poor handling of solid waste poses to both human health and the environmental safety, solid waste management has not been given proper attention in developing nations (Maiyaki et al., 2018). This statement supports the findings in the Kosmos study area whereby waste collection was the most identified problem, and points to the failure by local government to provide this service. Solid waste management is given low priority in developing countries because they are confronting other ‘more pressing’ and immediate challenges such as high infant mortality, staggering rates of HIV/AIDS cases and difficulties in providing basic amenities such as potable water and reliable energy sources (Tukahirwa et al., 2010). If waste is collected and transported, due to lack of sanitary landfills, the waste usually ends up at improper waste disposal sites where it poses a further hazard to the environment and human health (Katusiimeh et al., 2012). This practice perpetuates the high levels of environmental injustice in poor communities. Alleviating the chronic and acute human health and wellbeing problems in informal settlements is a key motivation for upgrading interventions and meeting the demand for environmental justice for all (French et al., 2021).

The Kosmos informal settlement is exposed and at greater risk from water related environmental consequences. Water availability has been part of the literature on sustainable development (Olaniyan et al., 2017) and is even cited in the Millennium Development Goals as an important resource to human life. The Kosmos informal settlement was categorised under the Full Level of Service (FLOS), and this is mainly due to its proximity to the private residential properties within the Kosmos area, and that the municipal water source passes through the informal settlement towards the private residences. This presented the settlement with some level of environmental justice in terms of water provision. Sustainable human development is premised upon the accessibility and availability of socio-economic services, equally important is sustainable access to water, which is intrinsically a backbone to life’s sustenance (Muzondi, 2014).

Water provision is universally accepted as the panacea for sustainable human development (Butuala et al., 2010) cited by Muzondi (2014). For justice to continuously prevail, there is a high demand for constant water supply in Kosmos informal settlement. Wats (2003) argued that the sustainability of water provision also calls for the adoption of proper service planning strategies and approaches in informal settlements. Traditionally, government agencies are vested with the responsibility of providing universal access to services such as water, sanitation, and electricity (Narayanan et al., 2017). Unfortunately, this has not been the case in the Kosmos study site and remains part of the environmental justice debates for informal settlement communities.

The provision of water has caught the attention of many scholars, and researchers continue to discuss it as one of the environmental justice matters that requires urgent attention. Water scarcity has been part of developmental challenges across the world and remains a serious challenge in Sub-Saharan Africa. The Millennium Development Goals, especially goals 7 and 8, state that any intervention in informal settlements must ensure environmental sustainability in human settlements which includes access to all adequate, safe, and affordable housing and basic services (Nassar & Elsayed, 2018). With the advent of Covid-19, Corona virus disease, the need for constant and continuous water supply cannot be overemphasized. Water, sanitation, and hygiene interventions reduce the incidence of water-borne and communicable diseases often yielding widespread health improvements for the whole community (Corban & Karanja, 2014). For many scholars, provision of health improving services is an important antidote for environmental justice.

## Conclusions

This paper presented environmental justice as one of the central but silent challenge that communities around the world continue to fight for. From the Aboriginal communities of India (McGregor, 2009), to the coastal communities of Cape Town (Jehanzaib et al., 2020), there seem to be a common need for communities to resist environmental injustices. The Kosmos case study findings and observations show that informal settlement’ challenges go far beyond the community’s ability to provide for themselves. This ability is also marred by many other pressing socio-economic needs such as health, food security, education for children, electricity, shelter, sanitation, water provision and security of tenure. The paper provides two lessons for the future of informal settlements and how to go about dealing with environmental injustices such as air pollution, sanitation, waste management and water provision facing communities in these settlements. Firstly, it exposes the notion that the government knows what the people want, and therefore, no need to consult them. There is also a general perception about the level of education for people living in informal settlements. They are often viewed as uneducated, backward, and generally have no sense of planning. Ntiwane and Coetzee (2018), maintains that there is need for a multi-stakeholder democratic planning procedure (PJ) that involves all stakeholders in the planning and implementation process, regardless of social structure and power, thereby providing equal access to deliberation, information, and consensus-building. Recognising the role of communities is important for EJ to prevail.

Provision of pit latrines in the Kosmos informal settlement that were subsequently not maintained was not only a waste of resources that could have been used to improve the sanitation facilities of the Kosmos community, but a failure by the government to provide basic services to the community. A community survey could have provided the municipality with a better and sustainable long-term sanitation solution for the informal settlement. The area has no running sewer to connect a water borne sanitation system, but the use of a common, well-built septic tank system is a possible long-term option and solution for the Kosmos informal settlement. This system is being used inside the private residence of Kosmos Village and some rural schools albeit with all its potential challenges, but it works. Improving sanitation in this regard will present some level of environmental justice to the Kosmos informal settlement community.

There is a large body of water behind the informal settlement (Hartbeespoortdaam), that could be utilised for a well-engineered sanitation wastewater system. The second lesson learned in this paper is the need to investigate challenges in informal settlements to allow an integrated sustainable solution to be delivered. Community participation and recognition remains at the centre of this integrated approach. Environmental justice remains a challenge for informal settlement communities and remains central to development initiatives. Poor communities still endue high environmental risks and remain subjected to poor living conditions. Distributive and social injustice emerged as prevalent in the Kosmos informal settlement. Decisions to support environmental justice for sustainable communities should incorporate the people’s voices, to ensure applicability and utilisation of any intervention. It is sometimes easier to use those affected to come up with solutions or to drive proposed solutions affecting their daily lives.

## Data Availability

The original contributions presented in the study are included in the article/supplementary material, further inquiries can be directed to the corresponding author.

## Appendix 1

See the Table

**Table 3:** Participants per stratum

Area	Age group	Actual sample	Percentage (%)
Kosmos	0–20	18	18/90 = 0.2
	21–40	42	42/90 = 0.4
	41–60	30	30/90 = 0.3

^3^.
